# Glucose-6-Phosphate Dehydrogenase::6-Phosphogluconolactonase from the Parasite *Giardia lamblia*. A Molecular and Biochemical Perspective of a Fused Enzyme

**DOI:** 10.3390/microorganisms9081678

**Published:** 2021-08-07

**Authors:** Laura Morales-Luna, Abigail González-Valdez, Beatriz Hernández-Ochoa, Roberto Arreguin-Espinosa, Daniel Ortega-Cuellar, Rosa Angélica Castillo-Rodríguez, Víctor Martínez-Rosas, Noemi Cárdenas-Rodríguez, Sergio Enríquez-Flores, Luis Miguel Canseco-Ávila, Verónica Pérez de la Cruz, Fernando Gómez-Chávez, Saúl Gómez-Manzo

**Affiliations:** 1 Laboratorio de Bioquímica Genética, Instituto Nacional de Pediatría, Secretaría de Salud, Ciudad de México 04530, Mexico; lauraeloisamorales@ciencias.unam.mx (L.M.-L.); ing_vicmr@hotmail.com (V.M.-R.); 2 Posgrado en Ciencias Biológicas, Universidad Nacional Autónoma de México, Ciudad de México 04510, Mexico; 3 Departamento de Biología Molecular y Biotecnología, Instituto de Investigaciones Biomédicas, Universidad Nacional Autónoma de México, Ciudad de México 04510, Mexico; abigaila@biomedicas.unam.mx; 4 Laboratorio de Inmunoquímica, Hospital Infantil de México Federico Gómez, Secretaría de Salud, Ciudad de México 06720, Mexico; beatrizhb_16@comunidad.unam.mx; 5 Departamento de Química de Biomacromoléculas, Instituto de Química, Universidad Nacional Autónoma de México, Ciudad de México 04510, Mexico; arrespin@unam.mx; 6 Laboratorio de Nutrición Experimental, Instituto Nacional de Pediatría, Secretaría de Salud 04530, Mexico; dortegadan@gmail.com; 7 Consejo Nacional de Ciencia y Tecnología (CONACYT), Instituto Nacional de Pediatría, Secretaría de Salud, Ciudad de México 04530, Mexico; racastilloro@conacyt.mx; 8 Laboratorio de Neurociencias, Instituto Nacional de Pediatría, Secretaría de Salud, Ciudad de México 04530, Mexico; noemicr2001@yahoo.com.mx; 9 Grupo de Investigación en Biomoléculas y Salud Infantil, Laboratorio de EIMyT, Instituto Nacional de Pediatría, Secretaría de Salud, Ciudad de México 04530, Mexico; sergioenriquez@ciencias.unam.mx; 10 Facultad de Ciencias Químicas, Campus IV, Universidad Autónoma de Chiapas, Tapachula 30798, Mexico; cansecoavila@gmail.com; 11 Neurochemistry and Behavior Laboratory, National Institute of Neurology and Neurosurgery “Manuel Velasco Suárez”, Ciudad de México 14269, Mexico; veped@yahoo.com.mx; 12 Laboratorio de Inmunología Experimental, Instituto Nacional de Pediatría, Ciudad de México 04530, Mexico; fergocha@gmail.com; 13 Cátedras CONACyT-Instituto Nacional de Pediatría, Secretaría de Salud, Ciudad de México 04530, Mexico; 14 Departamento de Formación Básica Disciplinaria, Escuela Nacional de Medicina y Homeopatía del Instituto Politécnico Nacional, Ciudad de México 07320, Mexico

**Keywords:** *Giardia lamblia*, glucose 6 phosphate dehydrogenase, drug target, metabolism, fused enzyme

## Abstract

*Giardia lamblia* is a single-celled eukaryotic parasite with a small genome and is considered an early divergent eukaryote. The pentose phosphate pathway (PPP) plays an essential role in the oxidative stress defense of the parasite and the production of ribose-5-phosphate. In this parasite, the glucose-6-phosphate dehydrogenase (G6PD) is fused with the 6-phosphogluconolactonase (6PGL) enzyme, generating the enzyme named G6PD::6PGL that catalyzes the first two steps of the PPP. Here, we report that the G6PD::6PGL is a bifunctional enzyme with two catalytically active sites. We performed the kinetic characterization of both domains in the fused G6PD::6PGL enzyme, as well as the individual cloned G6PD. The results suggest that the catalytic activity of G6PD and 6PGL domains in the G6PD::6PGL enzyme are more efficient than the individual proteins. Additionally, using enzymatic and mass spectrometry assays, we found that the final metabolites of the catalytic reaction of the G6PD::6PGL are 6-phosphoglucono-δ-lactone and 6-phosphogluconate. Finally, we propose the reaction mechanism in which the G6PD domain performs the catalysis, releasing 6-phosphoglucono-δ-lactone to the reaction medium. Then, this metabolite binds to the 6PGL domain catalyzing the hydrolysis reaction and generating 6-phosphogluconate. The structural difference between the *G. lamblia* fused enzyme G6PD::6PGL with the human G6PD indicate that the G6PD::6PGL is a potential drug target for the rational synthesis of novels anti-*Giardia* drugs.

## 1. Introduction

*G. lamblia* is a protist parasite causing giardiasis, a gastrointestinal disease. It is considered the most frequent parasitosis globally, and it is estimated that around 5 to 10% of the world population, which is approximately 300 million people, are affected by this microorganism [1,2,3]. Developing countries present the highest incidence, associating this disease with poorness and low health standards [4]. Giardiasis presents a high percentage of morbidity in children and the immunocompromised population [4]. Approximately 15% of children from 0 to 24 months of age in developing countries have developed this disease, which is associated with malabsorption syndrome and delayed cognition, which, in the most severe forms of the infection, can affect children’s physical and intellectual development [5].

**Figure 1:**
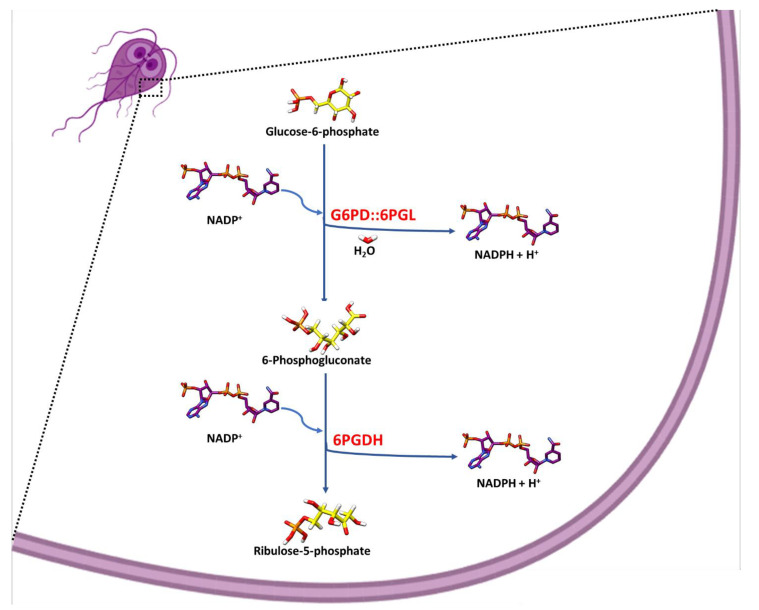
Proposed scheme of the Pentose Phosphate Pathway (PPP) oxidative phase from *G. lamblia*. A schematic representation of oxidative phase of PPP in organisms with fused glucose-6-phosphate dehydrogenase-6-phosphogluconolactonase (G6PD::6PGL) enzyme, and 6-phosphogluconate dehydrogenase (6PGDH) enzyme.

*G. lamblia* has a minimalistic genome, and many of the glycolytic and pentose phosphate pathway (PPP) enzymes share more similarities with prokaryote than eukaryote homologs [1]. PPP plays a crucial role in the parasite by generating the reduced nicotinamide-adenine-dinucleotide phosphate (NADPH). NADPH serves as an electron donor in biosynthetic processes and as an oxidative stress defense against the host. The PPP also provides ribose 5-phosphate (R5P) as a nucleic acid and various intermediate metabolites precursors such as fructose 6-phosphate (F6P) and glyceraldehyde 3-phosphate (G3P) [6]. In the PPP oxidative phase, the first reaction involving the oxidation of glucose 6-phosphate (G6P) to (6PGL) is catalyzed by the enzyme glucose-6-phosphate dehydrogenase (G6PD). Subsequently, the hydrolysis of 6-phosphoglucono-δ-lactone is mediated by the 6-phosphogluconolactonase (6PGL) to generate 6-phosphogluconate. The third reaction is the decarboxylation of 6-phosphogluconate by the 6-phosphogluconate dehydrogenase (6PGDH); then ribulose 5-phosphate and NADPH molecules are generated (Figure 1).

Interestingly, in *G. lamblia*, the enzymes mediating the first and the second reaction of the oxidative phase are fused (G6PD::6PGL); others have hypothesized that this fusion makes the oxidative phase more efficient (Figure 1) [7,8]. In a previous study, Morales-Luna et al. [9] suggested the presence of the structural NADP^+^ binding site in the G6PD region of the fused G6PD::6PGL enzyme in a 3D model from *G. lamblia*. However, only the G6PD domain has been characterized, and it is unknown if the 6PGL domain of the fused G6PD::6PGL enzyme is catalytically active.

This study aimed to kinetically characterize the 6PGL domain from the *G. lamblia* fused protein and the G6PD and the 6PGL separate proteins. The results showed that the G6PD and 6PGL domains in the fused G6PD::6PGL enzyme have more efficient catalysis concerning the individual proteins. Moreover, we determine by spectrophotometric and MALDI-TOF mass spectrometry methods that the metabolites 6-phosphoglucono-δ-lactone and 6-phosphogluconate are present as final products of catalytic reaction. The results suggest that the G6PD domain produces 6-phosphoglucono-δ-lactone and releases it to the reaction medium; the 6PGL domain takes this metabolite to catalyze its hydrolysis reaction generating 6-phosphogluconate. The structural and functional particularities of the bifunctional *G. lamblia* G6PD::6PGL enzyme regarding the human G6PD strongly suggest that this fused enzyme could be proposed as a new pharmacological target for the rational design of specific drugs for the treatment against this etiological agent.

## 2. Materials and Methods

### 2.1. Cloning of the Individual g6pd and 6pgl Gene Regions of the Fused g6pd::6pgl Gene from Giardia lamblia

From the sequence deposited in the Gene Data Bank (accession number GL50803_8682), specific oligonucleotides were designed to amplify the *g6pd*, and *6pgl* regions present in the sequence fused *g6pd*::*6pgl* gene from *G. lamblia*. The primers used for the amplification forward (Fw) and reverse (Rv) contain a sequence for restriction enzymes *Nde*I and *Bam*HI, respectively (Table S1). The amplification of the *g6pd* and *6pgl* regions was carried out by endpoint PCR using the plasmid pET3a-HisTEVP/*g6pd*::*6pgl*, which contained the fused gene *g6pd*::*6pgl* of *G. lamblia* [9].

The PCR reaction mix contained 200 ng of oligonucleotides, 200 ng of DNA as a template, ten mM of dNTP, 1X PCR buffer, and 1 U of Phusion enzyme^®^ High Fidelity DNA polymerase (Thermo Scientific, Waltham, MA, USA). Amplification conditions were: 1 min at 98 °C for denaturation, 30 cycles of amplification (30 s at 98 °C, 20 s at 65 °C, 30 s at 72 °C), and 5 min at 72 °C for the extension. The PCR product was separated using 1% agarose gel electrophoresis, stained with GelRed (Nucleic Acid Gel, Biotium, Fremon, CA, USA), and visualized in a MultiDoc-It Digital Imaging System (UVP, Upland, CA, USA).

The PCR products corresponding to fragments of interest were purified using the GeneJET Gel Extraction kit (Thermo Scientific, Waltham, MA, USA) and ligated into the pJET1.2/blunt vector (Thermo Scientific, Waltham, MA, USA). The resulting constructs were named pJET1.2/blunt-*g6pd* and pJET1.2/blunt-*6pgl* (Table S1) and were used to transform *E. coli* TOP10F’ competent cells. Subsequently, plasmid DNA of each construction was extracted using the GeneJET Plasmid Miniprep Kit (Thermo Scientific. Waltham, MA, USA) following the manufacturer’s instructions. First, to confirm the fidelity of *g6pd* and *6pgl* regions, bidirectional DNA sequencing with internal Fw and Rv sequencing primers was performed. Next, the verified sequences were digested with the restriction enzymes *Nde*I and *Bam*HI. Then, *g6pd* and *6pgl* regions were subcloned into the pET3a-HisTEV expression vector to obtain the plasmids named pET3a-HisTEVP/*g6pd* and pET3a-HisTEVP/*6pgl*, respectively (Table S1).

### 2.2. Expression and Purification of Fused G6PD::6PGL and Individual G6PD and 6PGL Proteins 

The expression of the recombinant fused G6PD::6PGL protein was performed as previously described by Morales-Luna et al. [8]. The *E. coli* BL21 (DE3)Δ*zwf*::kanr cells with the desired plasmids were grown for 12 h at 37 °C in 25 mL of Luria Bertani (LB) medium containing 100 µg/mL of ampicillin, and inoculated into 2 L of medium containing 100 µg/mL of ampicillin. The bacterial cultures were grown for 8 h at 37 °C and 160 rpm until they reached an optical density of 1.0 at 600 nm. Then the cultures were induced with 0.3 mM of isopropyl β-D-thiogalactopyranoside (IPTG) and incubated with shaking at 25 °C for 12 h. Cells were concentrated by centrifugation, washed with phosphate buffer (50 mM K_2_HPO_4_, 10% glycerol, 150 mM NaCl, at pH 7.35), and lysed by sonication using lysis buffer (50 mM K_2_HPO_4_, 10% glycerol, 150 mM NaCl, 0.5 mM PMSF, and 0.1% β-mercaptoethanol, at pH 7.35) as previously reported by Morales-Luna et al. [8,9]. The lysate was centrifuged at 13,000× *g* for 30 min to obtain the crude extract to purify the individual proteins.

The fused G6PD::6PGL protein and the individual 6PGL protein were purified using Ni Sepharose high-performance column previously equilibrated with equilibrium buffer (50 mM KH_2_PO_4_; 350 mM NaCl; glycerol 10%; pH 7.35) and incubated with the crude extract at 30 °C under stirring for 30 min. Then, the column was washed with equilibrium buffer plus 40 mM imidazole. Finally, the proteins were eluted with 250 mM imidazole in an equilibrium buffer. On the other hand, the individual G6PD protein was purified as previously described by Gomez-Manzo et al. [10,11]. To determine the presence of the fused G6PD::6PGL and the individual G6PD protein in the fractions, the activity of G6PD was determined spectrophotometrically by the reduction of the NADP^+^ at 340 nm and 25 °C with standard reaction mixture (0.1 M Tris-HCl buffer, 3 mM MgCl_2_, 200 µM G6P and 200 µM NADP^+^, pH 8.0). To determine the presence of individual 6PGL protein, the enzymatic activity was measured by a coupled assay using the enzyme 6-phosphogluconate dehydrogenase (6PGDH) from *G. lamblia* as a reporter enzyme, and the reduction of NADP^+^ was monitored at 340 nm in reaction buffer T (100 mM Tris-HCl buffer, 3 mM MgCl_2_, pH 8.0) with 300 µM 6-phosphoglucono-δ-lactone and 200 µM NADP^+^ [12]. The fractions with G6PD and 6PGL activity were concentrated using a microcon-10 kDa centrifugal filter unit Millipore (Millipore, Burlington, MA, USA). The purified proteins were used immediately in the later essays.

### 2.3. Kinetic Characterization of the Domains G6PD, 6PGL of the Fused G6PD::6PGL, and the Individuals G6PD and 6PGL Proteins

Kinetic characterization of the G6PD and 6PGL domains of the fused enzyme and the individual G6PD protein were determined. The initial velocities of the G6PD domain of the fused protein, as well as of the individual G6PD protein were obtained by varying the concentration of the substrate glucose 6-phosphate (G6P) or nicotinamide adenine dinucleotide phosphate (NADP^+^) in a range from 2.5 to 200 µM; while the second substrate was fixed at a saturation concentration (250 µM of NADP^+^ or G6P) in buffer T. The enzymatic reaction was carried out with 1 µg of fused protein G6PD::6PGL or 4 µg of individual G6PD protein [10,11].

To determine the kinetic parameters of the 6PGL domain of the fused enzyme, we perform a coupled assay using the enzyme 6-phosphogluconate dehydrogenase (6PGDH) from *G. lamblia* as a reporter enzyme, and the reduction of NADP^+^ was monitored at 340 nm. The initial velocities were obtained in the presence of 20 µg of 6PGDH and 1 µg of fused G6PD::6PGL protein. The substrate 6-phosphoglucono-δ-lactone was obtained through the enzymatic reaction of the recombinant G6PD enzyme of *Gluconacetobacter diazotrophicus* [13], and the product was titrated under standard conditions. The initial velocities of the 6PGL domain were obtained by varying the concentration of 6-phosphoglucono-δ-lactone substrate from 2.5 to 300 µM in buffer T. For both enzymes, the initial velocities were fitting the data to the Michaelis–Menten equation via non-linear regression calculations, and the steady-state kinetic parameters, *Km*, *kcat*, and *Vmax*, were obtained [10]. One international unit (IU) of G6PD or 6PGL enzymatic activity was defined as the enzyme required to produce 1 µmol of product per minute per mg of protein. All the assays were performed in triplicate.

### 2.4. Identification of the Metabolites as Final Products of Fused G6PD::6PGL Enzyme 

#### 2.4.1. Spectrophotometric Method

To determine if the 6-phosphogluconate is one of the metabolites present in the final enzymatic reaction of fused G6PD::6PGL enzyme, we perform a coupled assay with the 6PGDH enzyme and the final product of the reaction of fused G6PD::6PGL enzyme [12]. As a first step, the fused G6PD::6PGL enzyme was incubated with 100 µM of G6P and 100 µM of NADP^+^ in a final volume of 1 mL of the reaction mixture (0.1 M Tris-HCl, 3 mM MgCl_2_, pH 8.0), and the catalytic activity of G6PD region was monitored by the reduction of the NADP^+^ substrate at 340 nm in the Varian Cary 100 UV-VIS spectrophotometer (Agilent, Santa Clara, CA, USA). Later, the G6PD::6PGL enzyme was separated from the final reaction mixture using a Centricon-30 (Millipore, USA), and the final reaction mixture (900 µL) plus 100 µM of NADP^+^ was used to couple the reaction with the 6PGDH enzyme. The reaction was started with the addition of 1 µg of 6PGDH enzyme. The 6PGDH enzyme activity was detected through NADPH production at 340 nm in the Varian Cary 100 UV-VIS spectrophotometer (Agilent, Santa Clara, CA, USA), and the specific activity was calculated. Besides, the non-enzymatic reduction of NADP^+^ in the assay was measured and subtracted from the experimental points. The assay was performed in triplicate.

#### 2.4.2. MALDI-TOF Method 

The matrix-assisted laser desorption ionization time-of-flight mass spectrometry (MALDI-TOF MS) method was used to determine and identify the structures of the metabolites present in the final enzymatic reaction of fused G6PD::6PG enzyme. The final metabolites were obtained under the same conditions that in enzymatic methods. The final product was analyzed using the Microflex MALDI-TOF mass spectrometer (Bruker Daltonics, Bremen, Germany), and dihydroxybenzoic acid (DHB) was used as a matrix (Sigma Aldrich, St. Louis, MO, USA). For the identification of the metabolites, the obtained spectra were processed using the mMass analysis software, and comparison with the reference spectra present in the databases “The Human Metabolome database (www.hmdb.ca) and MassBank of North America (MoNA) (mona.fiehnlab.ucdavis.edu)” to identify the signals obtained with the signals previously reported for the compounds NADP^+^, G6P, 6-phosphoglucono-δ-lactone, and 6-phosphogluconate.

### 2.5. Prediction of the 6-Phosphoglucone-δ-lactone Substrate Binding Site 

To predicted the binding site of 6-phosphoglucono-δ-lactone on the 6PGL domain of the G6PD::6PGL from *G. lamblia*, the 3D model of G6PD::6PGL generated by Morales-Luna et al. [8] was aligned with the crystallographic structure of 6-phosphogluconolactonase from *Trypanosoma brucei* complexed with 6-phosphogluconic acid (PDB: 3E7F) [14]. To predict the atomic interactions of 6-phosphoglucono-δ-lactone on the G6PD::6PGL protein, blind docking was performed using the SwissDock Server (http://www.swissdock.ch/docking, accessed on 14 April 2021 [15]. Here, we used the 3D model of G6PD::6PGL from *G. lamblia* to add hydrogens to the structure. Then, the atomic coordinates were submitted to the MolProbity server (http://molprobity.biochem.duke.edu/, accessed 1 April 2021) [16], and system energy minimization was also performed using the YASARA force field [17]. The 3D structure of the 6-phosphoglucono-δ-lactone was downloaded from the PubChem server of the National Center of Biotechnology Information (PubChem CID:439452) (https://pubchem.ncbi.nlm.nih.gov/compound/439452, accessed on 1 April 2021). The docking was performed in the SwissDock server, which generates some possible binding modes of the ligand to the 3D model protein. Then, the most favorable binding modes at a given pocket were clustered. The predictions file provided Cluster Rank/Element Full Fitness and estimated binding free energy ΔG. In each experiment, was obtained a total of 256 poses per ligand. The generated docking results were loaded and analyzed into Chimera 1.15 software [18], and the affinity energies, the tridimensional configuration, the formation of hydrogen bonds, specific atoms involved, and the distance between them were analyzed to select the most favorable binding.

## 3. Results and Discussion 

### 3.1. Amplification and Cloning of the Individual g6pd and 6pgl Gene Regions of the Fused g6pd::6pgl Gene from G. lamblia 

**Figure 2:**
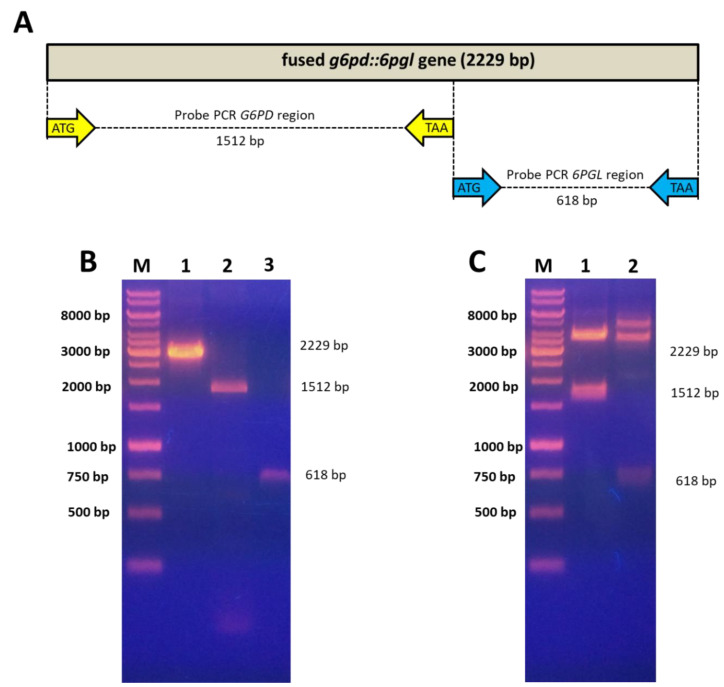
Amplification of the fused *g6pd*::*6pgl* gene and the individual *g6pd* and *6pgl* regions. (**A**) Schematic representation of fused *g6pd*::*6pgl* gene from *G. lamblia*, and the individual of *g6pd* and *6pgl* regions. (**B**) PCR products of amplification. Lane 1: PCR product of fused *g6pd*::*6pgl*. Lane 2: PCR product of the individual *g6pd* region. Lane 3: PCR product of the separate *6pgl* region. (**C**) Digestion of the pJET1.2/blunt-*g6pd* (lane 1), y pJET1.2/blunt-*6pgl* (lane 2) plasmids with N*de*I y *Bam*HI restriction enzymes. In both gels, GeneRuler 1Kb Plus DNA (Termo Scientifc^®^) was used as a molecular weight marker (M).

To corroborate if the fused enzyme G6PD::6PGL of *G. lamblia* is a bifunctional enzyme, we determine the kinetic parameters of the G6PD and 6PGL domains of fused enzyme and the individual G6PD protein. To obtain the individual G6PD and 6PGL proteins, the *g6pd* and *6pgl* regions of the fused *g6pd*::*6pgl* gene were individually amplified (Figure 2A). The amplification of *g6pd* and *6pgl* regions from the fused gene was performed by endpoint PCR. Figure 2B shows the PCR product corresponding to the fused *g6pd*::*6pgl* gene with a relative molecular size of 2229 base pairs (bp) (Figure 2B, lane 1); while the PCR product of the individual *g6pd* region has a relative size of 1512 bp (Figure 2B, lane 2). Finally, the PCR product belonging to the individual *6pgl* region with a relative size of 618 bp is shown (Figure 2B, lane 3). Then, the PCR products were cloned into the pJET1.2/Blunt vector and transformed into competent *E.coli* Top10F’ cells. The constructions were corroborated by digestion with the restriction enzymes *Nde*I and *Bam*HI. As seen in Figure 2C (lane 1), the digestion of plasmid pJET1.2/blunt-*g6pd* released a fragment of 1512 bp corresponding to the *g6pd* gene. While the plasmid pJET1.2/blunt-*6pgl* released a fragment around 618 bp, which corresponds to the *6pgl* gene (Figure 2C, lane 2).

### 3.2. Purification of Fused G6PD::6PGL and Individual G6PD and 6PGL Proteins 

**Figure 3:**
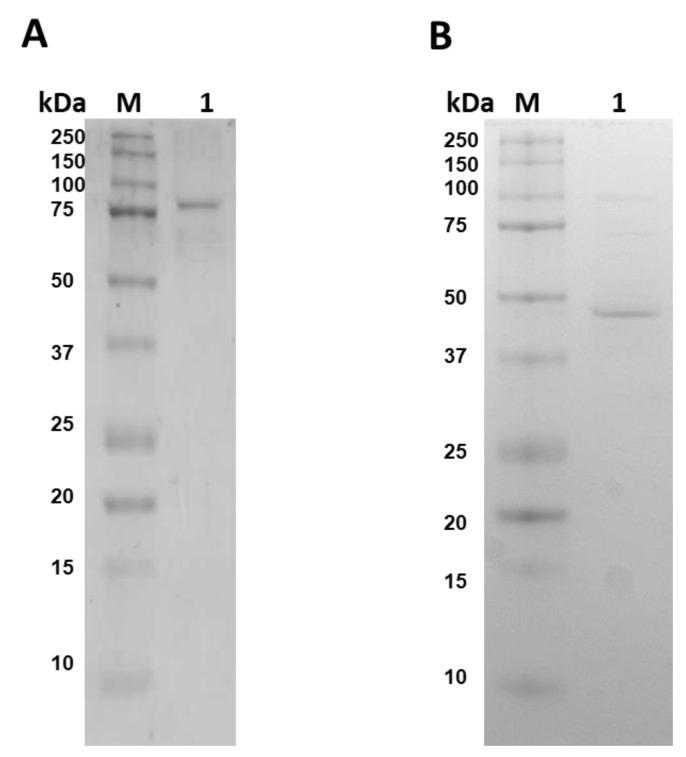
Purification of fused G6PD::6PGL and individual G6PD proteins. (**A**) Polyacrylamide gel electrophoresis SDS-PAGE (12%) of the recombinant fused G6PD::6PGL protein. Lane 1: 10 µg of purified G6PD::6PGL protein. (**B**) Polyacrylamide gel electrophoresis SDS-PAGE (12%) of the individual G6PD protein. Lane 1: 10 µg of purified individual G6PD protein. M: molecular protein weight (MW) marker precision plus protein kaleidoscope standards from Bio-Rad (Bio-Rad, Hercules, CA, USA). The gels were stained with colloidal Coomassie Brilliant Blue (R-250) (Sigma-Aldrich). The gels are representative of three individual experiments.

The fused G6PD::6PGL recombinant enzyme was purified to evaluate the catalytic activity of the G6PD and 6PGL domains and to identify the products present in the final reaction of enzymatic reaction. The fused enzyme was overexpressed and purified by Ni Sepharose high-performance column, and the purity was evaluated by 12% SDS-PAGE. As shown in Figure 3A, a single band is observed with a relative molecular weight (MW) of 82 kDa, which corresponds to the expected MW for the fused G6PD::6PGL enzyme. Table S2 shows the summary of the G6PD::6PGL purification, the total amount of protein of 2.8 mg/L of culture, a specific activity of 11.5 µmol·min^−1^·mg^−1^, and a yield of 11%.

On the other hand, the individual G6PD protein was purified, as seen in Figure 3B. The SDS-PAGE gel showed a predominant band with a relative MW of 56 kDa and was obtained 0.8 mg of total protein per 2 L of culture and a yield of 0.9% (Table S3). Besides, the specific activity determined was 0.121 µmol·min^−1^·mg^−1^. These results indicate that the purification of the individual G6PD protein presented a 10-fold lower yield than fused G6PD::6PGL enzyme (11%). In addition, we observed that the specific activity determined for the individual G6PD enzyme (0.121 µmol·min^−1^·mg^−1^) was 95-fold lower than the specific activity determined in the G6PD domain of the fused enzyme G6PD::6PGL (11.51 µmol·min^−1^·mg^−1^) [8]. We perform size exclusion chromatography of individual G6PD protein to evaluate the presence of aggregates in the purified protein. As seen in the gel filtration chromatogram; we only observed one peak (dimer of G6PD = 126 kDa) with G6PD activity (Figure S1A). This result indicates that when the G6PD is in the individual form, the protein is homogeneous and not aggregated (Figure S1B). This result is in concordance with the previously observed by Morales-Luna et al. [8], where a dimer active in solution was obtained for the fuse G6PD::6PGL enzyme. 

Finally, the individual 6PGL protein purification was not achieved using our methodology, and the enzymatic activity for individual 6PGL protein could not be determined. In this context, it is important to mention that previously in our working group, to explore the stability of the G6PD::6PGL structure, a Molecular Dynamics (MD) Simulation of the fused G6PD::6PGL enzyme was performed. As a result, we observed that according to the root mean square deviation (RMSD) and the radius of gyration (Rg), when the cofactor NADP^+^ was added, the fluctuations decreased, indicating that NADP^+^ had a stabilizing effect, especially on the 6PGL domain. Besides, when only structural NADP^+^ was present, the stabilization of 6PGL was more evident than that produced by cofactor NADP^+^ since fluctuations were minor [8].

### 3.3. Kinetic Characterization of the Domains G6PD, 6PGL of the Fused G6PD::6PGL, and the Individual G6PD Protein 

**Figure 4:**
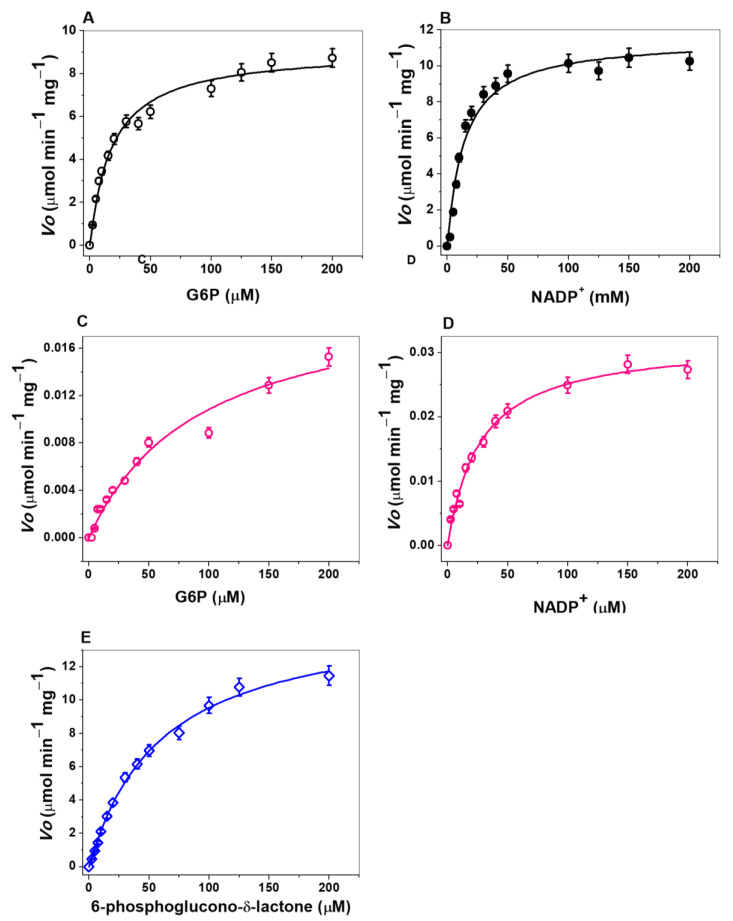
Michaelis–Menten plots for G6PD domain of fused G6PD::6PGL enzyme with (**A**) G6P and (**B**) NADP^+^ as substrates. Michaelis–Menten plots for individual G6PD protein with (**C**) G6P and (**D**) NADP^+^ as substrates. Michaelis–Menten plots for 6PGL domain of fused G6PD::6PGL with (**E**) 6-phosphoglucono-δ-lactone substrate. The data represent the mean ± SD from five independent experiments.

The kinetic parameters of the G6PD and 6PGL domains of the fused G6PD::6PGL enzyme and the individual G6PD protein were determined. As seen in Figure 4, Michaelis–Menten behavior of the G6PD::6PGL (Figure 4A,B) and individual G6PD (Figure 4C,D) enzymes were observed for both substrates, G6P and NADP^+^. Thus, the initial velocity values were fitted to the Michaelis–Menten equation by non-linear regression calculations. To obtain the initial velocities for individual G6PD protein is important to mention that it was necessary to add 4-fold more protein (4 µg total) to start the reaction concerning the amount used in the saturation curves for the fused G6PD::6PGL enzyme (1 µg total). Finally, Figure 4E shows the Michaelis–Menten plots for the 6PGL domain of the fused G6PD::6PGL enzyme. In all cases, a Michaelis-Menten behavior was observed for the substrates tested. Finally, the steady-state kinetic parameters were obtained (Table 1). The K_m_ values for the G6PD domain of the fused enzyme were K_m_ G6P = 18.1 µM and K_m_ for NADP^+^ = 13.9 µM; while for the individual G6PD protein, a value of K_m_ G6P = 94.2 µM and K_m_ NADP^+^ = 26.7 µM was determined. Furthermore, the K_m_ values determined for the individual G6PD protein were 5-folds higher for the G6P substrate, while the NADP^+^ substrate increased 2-folds compared to that obtained in the fused G6PD::6PGL enzyme. These results demonstrate that there is a lower affinity for both substrates in the individual G6PD protein. Similar results were reported for the individual G6PD protein of the fused G6PD::6PGL enzyme from *Plasmodium falciparum* (PfGluPhos) where the G6PD domain in this enzyme presented better affinity for the substrate G6P (19.2 mM) concerning the purified individual G6PD protein (33.2 µM). However, the same affinity for the catalytic NADP^+^ coenzyme was reported in the fused and individual enzymes (6.5 µM and 6.1 µM, respectively).

Regarding the k_cat_ value, a value of 0.057 s^−1^ was obtained for the individual G6PD protein, which is 550 times lower than that obtained for the G6PD domain on the fused G6PD::6PGL enzyme (31.8 s^−1^). This result agrees with that previously observed in the fused PfGluPhos enzyme, where the G6PD and 6PGL domains were catalytically characterized, and a decreased catalytic activity was observed in the G6PD and 6PGL enzymes when these were individually characterized [19]. However, in the fused (PvGluPho) protein from *Plasmodium vivax* [20], the same specific activity was observed in the PvGluPho G6PD domain and the individually G6PD protein.

Finally, we determine only the kinetic parameters of the 6PGL domain of the fused G6PD::6PGL enzyme because the individual 6PGL protein could not be purified. As seen in Figure 4E, a Michaelis-Menten behavior is observed for the 6-phosphoglucono-δ-lactone substrate. A K_m_ of 51.5 µM was obtained for the substrate 6-phosphoglucono-δ-lactone, and a k_cat_ of 31.8 s^−1^. These results differ to other reported for 6PGLs from different organisms such as *Homo sapiens* (K_m_ = 242 µM,), *P. falciparum* (K_m_ = 172 µM), and *Saccharomyces cerevisiae* (K_m_ = 43 µM). Besides, an important finding is that for both G6PD and 6PGL domains of fused G6PD::6PGL *G. lamblia* enzyme, we obtained the same k_cat_ value = 31.8 s^−1^, which could indicate that both domains work in equilibrium since they have the same turnover number per unit of time when they are fused in the G6PD::6PGL enzyme.

In addition, when comparing the specificity constants for both enzymes in both G6P and NADP^+^ substrates, we found less efficiency for the individual G6PD enzyme for G6P (k_cat_/Km = 1.75 × 10^6^ s^−1^·M^−1^) and NADP^+^ (k_cat_/K_m_ = 2.29 × 10^6^ s^−1^·M^−1^) concerning the fused enzyme, for G6P (k_cat_/K_m_ = 6 × 10^2^ s^−1^·M^−1^) and NADP^+^ (k_cat_/K_m_ = 2.1 × 10^2^ s^−1^·M^−1^) which again indicates that the G6PD enzyme must be folded together with the 6PGL domain to perform higher efficient catalysis, which is the feasible flow of the pathway.

### 3.4. Identification of the Metabolites as Final Products of Fused G6PD::6PGL Enzyme

Bifunctional enzymes generally contain two large structural domains catalytically active on the same polypeptide chain whose association facilitates metabolic pathway control and/or allows more efficient substrate conversion [21]. In previous work, the catalytic activity of the G6PD domain of the fused G6PD::6PGL enzyme from *G. lamblia* was established [8], but until then, it was unknown if the 6PGL domain was catalytically active. To determine the catalytic activity of the 6PGL domain and thus the bifunctionality of the fused G6PD::6PGL enzyme, the final product of the catalytic reaction was analyzed by two different methods: (1) coupling of the final product of the reaction with an enzymatic method, and (2) MALDI-TOF mass spectrometry, allowing us to determine and identify the metabolites present in the final product of the reaction of the fused G6PD::6PGL enzyme.

#### 3.4.1. Enzymatic Methods 

**Figure 5:**
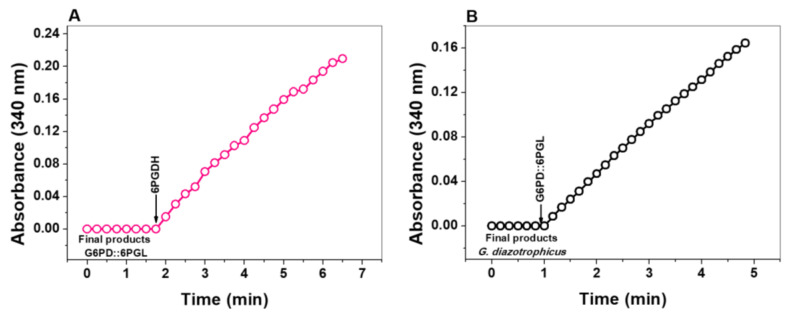
Determination of the catalytic activity of the 6PGL domain of the fused protein by coupled assay. (**A**) Evaluation of the final product of the catalysis of the fused G6PD::6PGL enzyme by coupled assay with the 6PGDH enzyme from *G. lamblia*. (**B**) The final product of the enzymatic reaction is catalyzed by the recombinant G6PD enzyme from *G. diazotrophicus* and coupled with the fused G6PD::6PGL enzyme.

We characterized the catalytic activity of the 6PGL domain by a coupled assay using the 6PGDH from *G. lamblia* as a reporter enzyme [12]. As seen in Figure 5A, when the final product of the fused G6PD::6PGL enzyme was incubated with the purified 6PGDH enzyme of *G. lamblia*, an increase in absorptivity was observed at 340 nm. This is due to NADPH production by the 6PGDH enzyme, which binds its physiological substrate (6-phosphogluconate) generated by the fused G6PD::6PGL enzyme. Besides, we evaluated if the 6PGL domain can bind the 6-phosphoglucono-δ-lactone substrate from the reaction medium. For this purpose, the 6-phosphoglucono-d-lactone substrate was produced using the recombinant G6PD enzyme from *Gluconacetobacter diazotrophicus* (GdG6PD) [13]. As seen in Figure 5B, an increase in absorptivity was observed at 340 nm when the fused G6PD::6PGL enzyme was incubated with the final product of the GdG6PD enzyme. These experiments confirmed that the 6PGL domain of the fused G6PD::6PGL enzyme is catalytically active and can take the substrate (6-phosphoglucono-δ-lactone produced by GdG6PD) from the reaction medium.

#### 3.4.2. MALDI-TOF Method 

**Figure 6:**
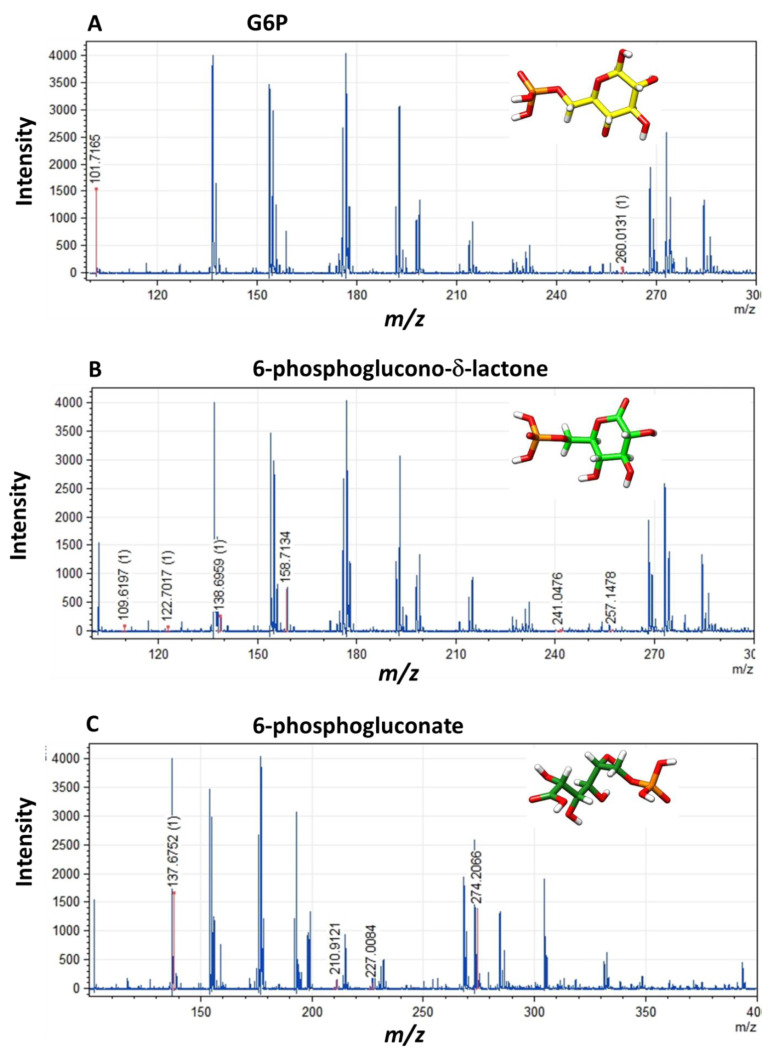
Mass spectra of the metabolites identified in the final product of the catalytic reaction of the fused G6PD::6PGL enzyme by MALDI-TOF MS. (**A**) Mass spectrum of the substrate glucose 6-phosphate, (**B**) 6-phosphoglucono-δ-lactone, (**C**) 6-phosphogluconate. Each peak corresponds to the masses of the fragmented metabolites ions present in the reaction of the G6PD::6PGL enzyme from *G. lamblia*. The experiment was performed in triplicate.

To determine if the 6-phosphoglucono-δ-lactone is present in the medium of the reaction as the final product of the catalysis performed by the fused enzyme, or if all glucose-6-phosphate molecules are catalyzed to 6-phosphogluconate, the final product of the reaction was analyzed by MALDI-TOF MS to determine and identify the structures of the metabolites present in the reaction mixture. The application of MALDI-TOF MS and the database used allowed the correct identification of the glucose 6-phosphate, 6-phosphoglucono-δ-lactone, 6-phosphogluconate, NADPH, and NADP^+^. The obtained mass spectra are shown in Figure 6, and the peaks list detected from the mass spectra is provided in Table 2. When the metabolites were identified in the reaction product of the fused enzyme, the substrate glucose 6-phosphate was identified at *m*/*z* 260.01, with a fragmented peak at *m*/*z* 101.71 (Figure 6A). For 6-phosphoglucono-δ-lactone, seven peaks of the fragmented ions were detected Figure 6B, the 6-phosphoglucono-δ-lactone was detected at *m*/*z* 257.14. While for 6-phosphogluconate (products of the 6PGL domain), four peaks were detected (Figure 6C) and the metabolite was detected at *m*/*z* 274.20. Besides, we observed peaks that correspond to the expected fragmented ions for NADP^+^ and NADPH (data not shown).

### 3.5. Identification of the 6-Phosphoglucono-δ-lactone Substrate Binding Site on G6PD::6PGL from G. lamblia 

**Figure 7:**
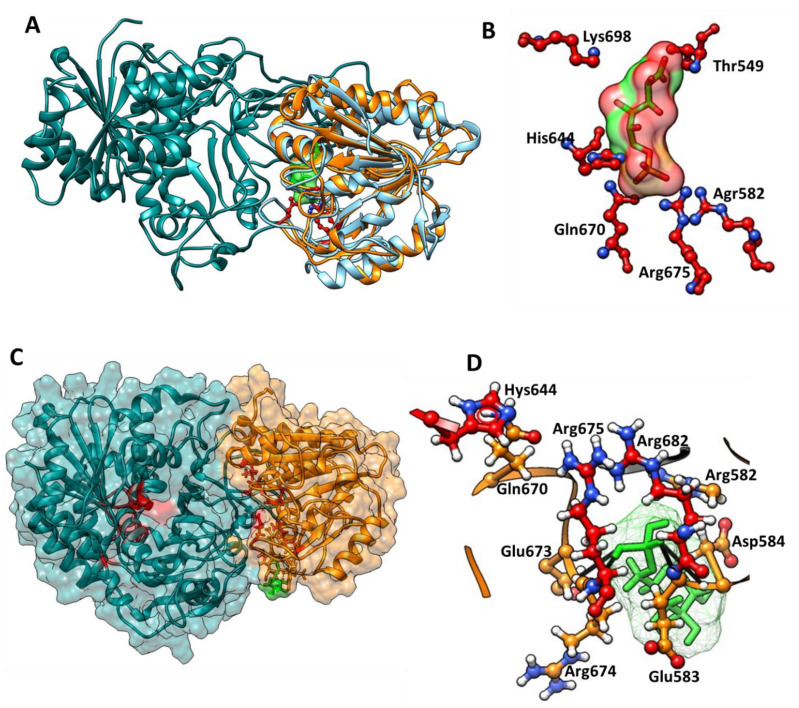
Identification of the 6-phosphoglucono-δ-lactone substrate binding site in G6PD::6PGL from *G. lamblia*. (**A**) Structural alignment of the 6PGL domain of the G6PD::6PGL (dark cyan::orange) with 6PGL of *T. brucei* (blue light). (**B**) Close-up of the substrate with keys amino acids. The phosphogluconic acid is shown in green color and the amino acids in red color. (**C**) Docking prediction between G6PD::6PGL protein from *Giardia lamblia* with 6-phosphoglucono-δ-lactone. A general view of the binding affinities of the zone of interaction near the catalytic site of the 6PGL domain. (**D**) Zoom of the zone of interaction of 6-phosphoglucono-δ-lactone (green color) with the amino acids to the binding pocket near the catalytic site of the 6PGL domain. The G6PD is shown in dark cyan color, the 6PGL domain is showed in orange color, and the amino acids involved in binding and catalysis are shown in red color.

To predict the amino acids involved in the binding site of 6-phosphoglucono-δ-lactone on the 6PGL domain of the G6PD::6PGL from *G. lamblia*, the 3D model of the G6PD::6PGL was aligned with the crystallographic structure of 6-phosphogluconolactonase from *Trypanosoma brucei* complexed with 6-phosphogluconic acid (PDB: 3E7F) [14]. The Tb6PGL showed a 27% sequence identity with the G6PD::6PGL from *G. lamblia*. The alignment and the root mean square deviation (RMSD) value of 1.647 Å was obtained for 202 Cα atoms, and the Q-score value was 0.563 (Figure 7A). For 6-phosphoglucono-δ-lactone, the key binding site residues Thr549, Arg582, His644, Gln670, Arg675, and Lys 698 were selected (Figure 7B).

Later, a molecular blind docking analysis was carried out to explore the binding affinities of 6-phosphoglucono-δ-lactone with the G6PD::6PGL from *G. lamblia*. The in silico study revealed one zone of interaction near the catalytic site of the 6PGL domain (Figure 7C); in this pocket, 9 amino acids residues were identified that interact with 6-phosphoglucono-δ-lactone, Arg582, Glu583, Asp584, His644, Gln670, Glu673, Arg674, Arg675, Arg682 (the amino acid number corresponds to the G6PD::6PGL sequence from *G. lamblia*; Figure 7D). Also, the docking analysis showed that four hydrogen bonds are formed between the phosphate group of the substrate and the amino acids Glu583, Asp584, and Arg674 (Figure 7D), and the most stable conformer presented a ΔG = −7.28 kcal/mol.

It is essential to mention that even though the computational modeling of proteins and the prediction of interactions with ligands has provided a broader vision in structural biology, complementing the experimental techniques. Such computational techniques still face some challenges and limitations, for example, the expected precision in the results and the structural variability of a specific model. Furthermore, considering that the function of a protein almost always involves movements and conformational changes, a molecular understanding of its mechanism requires a detailed description of the different dynamic functional states.

**Figure 8:**
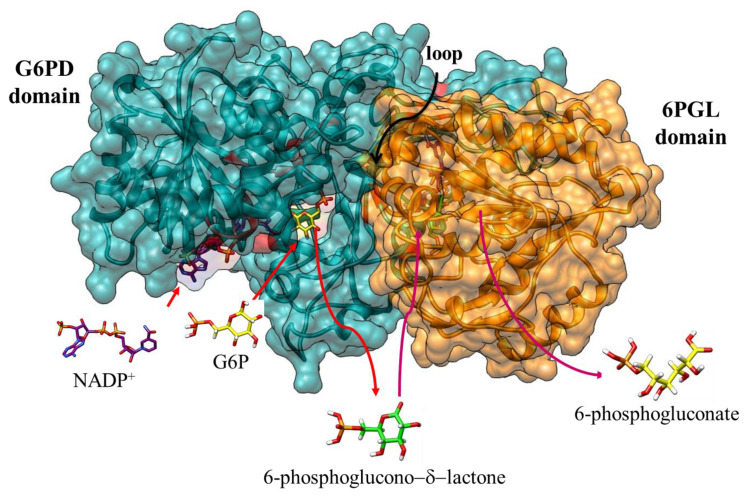
Catalysis of the fused G6PD::6PGL enzyme from *G. lamblia*. The surface representation of the G6PD domain is colored green, and the 6PGL domain is colored orange. The cofactor NADP^+^ (red color), and the substrate G6P (yellow color) and 6-fosfoglucono-d-lactone (green color) are shown as stick models. The arrows in red indicate the entry of the G6P and NADP^+^ substrates and the active site of the G6PD domain. The magenta arrows indicate the exit of the 6-phosphoglucono-δ-lactone product into the reaction medium and its entry into the active site of the 6PGL domain marked in orange color. Finally, the exit of the 6-phosphogluconate into the reaction medium is indicated with an arrow in magenta color.

Our results allow us to infer that individual G6PD and 6PGL regions need to be in their fused form to perform their physiological function efficiently. Besides, the data obtained through the coupled assay and mass spectrometry indicate that 6-phosphoglucono-δ-lactone and 6-phosphogluconate are present in the final product of the catalysis of the fused enzyme, so it is possible to infer that the fused G6PD::6PGL enzyme from *G. lamblia* is bifunctional because its two domains are catalytically active. Based on these results, we propose that the G6PD domain catalyzes its physiological substrates (G6P and NADP^+^), releasing the 6-phosphoglucono-δ-lactone and NADPH products to the reaction medium. Subsequently, the 6PGL domain takes the physiological substrate 6-phosphoglucono-δ-lactone from the reaction medium to bind at the catalytic site and hydrolyze it to 6-phosphogluconate, a metabolite that is finally released into the reaction medium (Figure 8). Furthermore, no substrate channeling (direct transfer between catalytic sites) was observed in the fused G6PD::6PGL enzyme. We consider that the G6PD domain does not directly transfer the product to the 6PGL domain, probably due to a spatial impediment (loop) that does not favor the direct passage of the G6PD domain at the active site of the 6PGL domain as seen in the model of the fused G6PD::6PGL enzyme (Figure 8). However, although no substrate channeling was observed, the bifunctional arrangement conveys a kinetic advantage owing to proximity effects (reduced soluble phase diffusion) [21].

## 4. Conclusions

In summary, we determined the catalytic parameters of the G6PD and 6PGl domains of the fused enzyme G6PD::6PGL and the individual G6PD. The individual G6PD protein showed lower catalytic activity and affinity for the physiological substrates G6P and NADP^+^ in the individual domain. This could result from a different folding in the individual G6PD protein that does not adequately bind the physiological substrates G6P and NADP^+^. Finally, the products of the fused enzyme were determined by coupled enzymatic assay and mass spectrometry, where 6-phosphoglucono-δ-lactone, 6-phosphogluconate, and NADPH were identified as final products of the catalytic activity. Furthermore, the proximity effect could possibly explain a higher catalytic effect with the intermediate product on the bifunctional G6PD::6PGL enzyme from *G. lamblia*, but this was not possible to test. 

## Figures and Tables

**Table 1:** Steady-state kinetic parameters of the fused G6PD::6PGL from *G. lamblia* and the individual proteins.

Kinetic Parameters	G6PD::6PGL		Individual Proteins	
	G6PD	6PGL	G6PD	6PGL
K_m_ G6P (µM)	18.1	−	31.8	n.d
K_m_ NADP^+^ (µM)	13.9	−	26.7	n.d
K_m_ Lactone (µM)	−	51.5	−	n.d
K_cat_ (s^−1^)	31.8	31.8	0.05	n.d

n.d = not determined.

**Table 2:** Expected and obtained mass determined by MADI-TOF for the catalytic reaction performed by the fused G6PD::6PGL enzyme from. *G. lamblia*.

Name Substrates/Products	Elemental Formula	Molecular Weight	Expected *m*/*z*	Obtained *m*/*z*
Glucose 6-phosphate	C_6_H_13_O_9_P	260.14	101, 102, 132, 194, 209, 211, 226, 256, 260	260.01, 101.71
6-phosphoglucono−δ−lactone	C_6_H_11_O_9_P	258.12	96, 98, 109, 122, 131, 138, 159, 171, 241, 257, 275	257.14, 241.04, 158.71, 138.69, 122.70, 109.61
6-phosphogluconate	C_6_H_12_O_10_P	275.12	104, 114, 125, 131, 137, 144, 153, 211, 227, 274	274.20, 227.00, 210.91, 237.67
NADPH	C_21_H_30_N_7_O_17_P_3_	745.4	123, 158, 166, 195, 257, 272, 287, 335, 415, 426, 471, 505, 520, 592, 728	158, 166, 272, 287, 335
NADP^+^	C_21_H_29_N_7_O_17_P_3_	744.41	122, 164, 194, 223, 246, 260, 272, 333, 426, 469, 505, 547, 562, 578, 580, 713, 725	194, 246, 260, 272, 333

## Supplementary Materials

The following are available online at https://www.mdpi.com/article/10.3390/microorganisms9081678/s1, Figure S1: Native status of the individual G6PD protein, Table S1: Strains, plasmids, and primers used in amplification of the individual *g6pd* and *6pgl* regions of the fused *g6pd*::*6pgl* gene from *Giardia lamblia*, Table S2: Purification of fused G6PD::6PGL protein, Table S3: Purification of the individual G6PD protein.

## References

[1] Morrison H.G., McArthur A.G., Gillin F.D., Aley S.B., Adam R.D., Olsen G.J., Best A.A., Cande W.Z., Chen F., Cipriano M.J. (2007). Genomic minimalism in the early diverging intestinal parasite *Giardia lamblia*. Science.

[2] Cernikova L., Faso C., Hehl A.B. (2018). Five facts about *Giardia lamblia*. PLoS Pathog..

[3] Escobedo A.A., Hanevik K., Almirall P., Cimerman S., Alfonso M. (2014). Management of chronic Giardia infection. Expert Rev. Anti-Infect. Ther..

[4] Cedillo-Rivera R., Darby J.M., Enciso-Moreno J.A., Ortega-Pierres G., Ey P.L. (2003). Genetic homogeneity of axenic isolates of *Giardia intestinalis* derived from acute and chronically infected individuals in Mexico. Parasitol. Res..

[5] Berkman D.S., Lescano A.G., Gilman R.H., Lopez S.L., Black M.M. (2002). Effects of stunting, diarrhoeal disease, and parasitic infection during infancy on cognition in late childhood: A follow-up study. Lancet.

[6] Duffieux F., Van Roy J., Michels P.A., Opperdoes F.R. (2000). Molecular characterization of the first two enzymes of the pentose-phosphate pathway of *Trypanosoma brucei*. Glucose-6-phosphate dehydrogenase and 6-phosphogluconolactonase. J. Biol. Chem..

[7] Stover N.A., Dixon T.A., Cavalcanti A.R. (2011). Multiple independent fusions of glucose-6-phosphate dehydrogenase with enzymes in the pentose phosphate pathway. PLoS ONE.

[8] Morales-Luna L., Serrano-Posada H., Gonzalez-Valdez A., Ortega-Cuellar D., Vanoye-Carlo A., Hernandez-Ochoa B., Sierra-Palacios E., Rufino-Gonzalez Y., Castillo-Rodriguez R.A., Perez de la Cruz V. (2018). Biochemical Characterization and Structural Modeling of Fused Glucose-6-Phosphate Dehydrogenase-Phosphogluconolactonase from *Giardia lamblia*. Int. J. Mol. Sci..

[9] Morales-Luna L., González-Valdez A., Sixto-López Y., Correa-Basurto J., Hernández-Ochoa B., Cárdenas-Rodríguez N., Castillo-Rodríguez R.A., Ortega-Cuellar D., Arreguin-Espinosa R., Pérez de la Cruz V. (2020). Identification of the NADP^+^ structural binding site and coenzyme effect on the fused G6PD:: 6PGL protein from *Giardia lamblia*. Biomolecules.

[10] Gómez-Manzo S., Terrón-Hernández J., la Mora-De la Mora D., González-Valdez A., Marcial-Quino J., García-Torres I., Vanoye-Carlo A., López-Velázquez G., Hernández-Alcántara G., Oria-Hernández J. (2014). The stability of G6PD is affected by mutations with different clinical phenotypes. Int. J. Mol. Sci..

[11] Gómez-Manzo S., Marcial-Quino J., Vanoye-Carlo A., Enríquez-Flores S., la Mora-De la Mora D., González-Valdez A., García-Torres I., Martínez-Rosas V., Sierra-Palacios E., Lazcano-Pérez F. (2015). Mutations of glucose-6-phosphate dehydrogenase Durham, Santa-Maria and A+ variants are associated with loss functional and structural stability of the protein. Int. J. Mol. Sci..

[12] Morales-Luna L., Hernandez-Ochoa B., Martinez-Rosas V., Gonzalez-Valdez A., Cardenas-Rodriguez N., Enriquez-Flores S., Marcial-Quino J., Gomez-Manzo S. (2021). Cloning, purification, and characterization of the 6-phosphogluconate dehydrogenase (6 PGDH) from *Giardia lamblia*. Mol. Biochem. Parasitol..

[13] Ramirez-Nava E.J., Ortega-Cuellar D., Gonzalez-Valdez A., Castillo-Rodriguez R.A., Ponce-Soto G.Y., Hernandez-Ochoa B., Cardenas-Rodriguez N., Martinez-Rosas V., Morales-Luna L., Serrano-Posada H. (2019). Molecular Cloning and Exploration of the Biochemical and Functional Analysis of Recombinant Glucose-6-Phosphate Dehydrogenase from *Gluconoacetobacter diazotrophicus* PAL5. Int. J. Mol. Sci..

[14] Duclert-Savatier N., Poggi L., Miclet E., Lopes P., Ouazzani J., Chevalier N., Nilges M., Delarue M., Stoven V. (2009). Insights into the enzymatic mechanism of 6-phosphogluconolactonase from *Trypanosoma brucei* using structural data and molecular dynamics simulation. J. Mol. Biol..

[15] Grosdidier A., Zoete V., Michielin O. (2011). SwissDock, a protein-small molecule docking web service based on EADock DSS. Nucleic Acids Res..

[16] Williams C.J., Headd J.J., Moriarty N.W., Prisant M.G., Videau L.L., Deis L.N., Verma V., Keedy D.A., Hintze B.J., Chen V.B. (2018). MolProbity: More and better reference data for improved all-atom structure validation. Protein Sci..

[17] Krieger E., Joo K., Lee J., Lee J., Raman S., Thompson J., Tyka M., Baker D., Karplus K. (2009). Improving physical realism, stereochemistry, and side-chain accuracy in homology modeling: Four approaches that performed well in CASP8. Proteins Struct. Funct. Bioinform..

[18] Pettersen E.F., Goddard T.D., Huang C.C., Couch G.S., Greenblatt D.M., Meng E.C., Ferrin T.E. (2004). UCSF Chimera--a visualization system for exploratory research and analysis. J. Comput. Chem..

[19] Jortzik E., Mailu B.M., Preuss J., Fischer M., Bode L., Rahlfs S., Becker K. (2011). Glucose-6-phosphate dehydrogenase-6-phosphogluconolactonase: A unique bifunctional enzyme from *Plasmodium falciparum*. Biochem. J..

[20] Haeussler K., Berneburg I., Jortzik E., Hahn J., Rahbari M., Schulz N., Preuss J., Zapol’skii V.A., Bode L., Pinkerton A.B. (2019). Glucose 6-phosphate dehydrogenase 6-phosphogluconolactonase: Characterization of the *Plasmodium vivax* enzyme and inhibitor studies. Malar. J..

[21] d Moore B. (2004). Bifunctional and moonlighting enzymes: Lighting the way to regulatory control. Trends Plant Sci..

