# Supplementary material for: Glucose-6-Phosphate Dehydrogenase::6-Phosphogluconolactonase from the Parasite Giardia lamblia. A Molecular and Biochemical Perspective of a Fused Enzyme
Source: Microorganisms. 2021 Aug 7;9(8):1678. doi: 10.3390/microorganisms9081678 (PMC8399836; doi:10.3390/microorganisms9081678)
Supplement: Supplementary file 1 [file microorganisms-09-01678-s001.zip › microorganisms-1325219-supplementary.pdf]

Table S1. Strains, plasmids, and primers used in amplification of the individual *g6pd* and *6pgl* regions of the fused *g6pd::6pgl* gene from *Giardia lamblia*.

**Bacterial strains**	
**Strain**	**Characteristics**
Top10F′	*Escherichia coli* competent cells.
BL21(DE3)Δ*zwf*::kan^r	*Escherichia coli* BL21(DE3)Δ*zwf*::kan^r cells with knout of the endogenous *zwf* gene.
**Plasmids**	
**Plasmid**	**Characteristics**
pJET1.2/blunt-*g6pd*	pJET1.2/Blunt containing the *g6pd* region of fused *g6pd::6pgl* gene from *Giardia lamblia* with ampicillin resistance.
pJET1.2/blunt-*6pgl*	pJET1.2/Blunt containing the *6pgl* region of fused *g6pd::6pgl* gene from *Giardia lamblia* with ampicillin resistance.
pET3a-HisTEVP/*g6pd::6pgl*	pET3a-HisTEVP containing the fused *g6pd::6pgl* gene from *Giardia lamblia* encoding the fused G6PD::6PGL protein. Plasmid with ampicillin resistance.
pET3a-HisTEVP-*g6pd*	pET3a-HisTEVP containing the *g6pd* region of fused *g6pd::6pgl* gene from *Giardia lamblia* encoding the individual G6PD protein. Plasmid with ampicillin resistance.
pET3a-HisTEVP-*6pgl*	pET3a-HisTEVP containing the *6pgl* region of fused *g6pd::6pgl* gene from *Giardia lamblia* encoding the individual 6PGL protein. Plasmid with ampicillin resistance.
**Oligonucleotides**	
Cloning oligonucleotides	Sequence
g6pd Fw	5′-GCGTCATATGTTCAAGCCTTCCTGC-3′
g6pd Rv	5′-CTGGGGATCCTTAATTGAACACTGG-3′
6pgl Fw	5′-GCGGGATCCTTAGTATATGATGGGCTT-3′
6pgl Rev	5′-GCGCCATATGAATAATAGCTTTGACATA-3′

Table S2. Purification of fused G6PD::6PGL protein.

**Step**	**Total protein (mg)**	**Specific activity (µmol·min^{-1}·mg^{-1})**	**Total activity (IU)**	**Yield (%)**
Crude extract	1638.0	0.00071	1.170	100.0
Q-Sepharose	386.4	0.00072	0.279	23.9
2′5 ADP-Sepharose	0.87	0.1217	0.010	0.9

Table S3. Purification of the individual G6PD protein.

**Step**	**Total protein (mg)**	**Specific activity (µmol·min^{-1}·mg^{-1})**	**Total activity (IU)**	**Yield (%)**
Crude extract	432.6	0.68	297.38	100
Ni-Sepharose	2.8	11.51	32.22	11

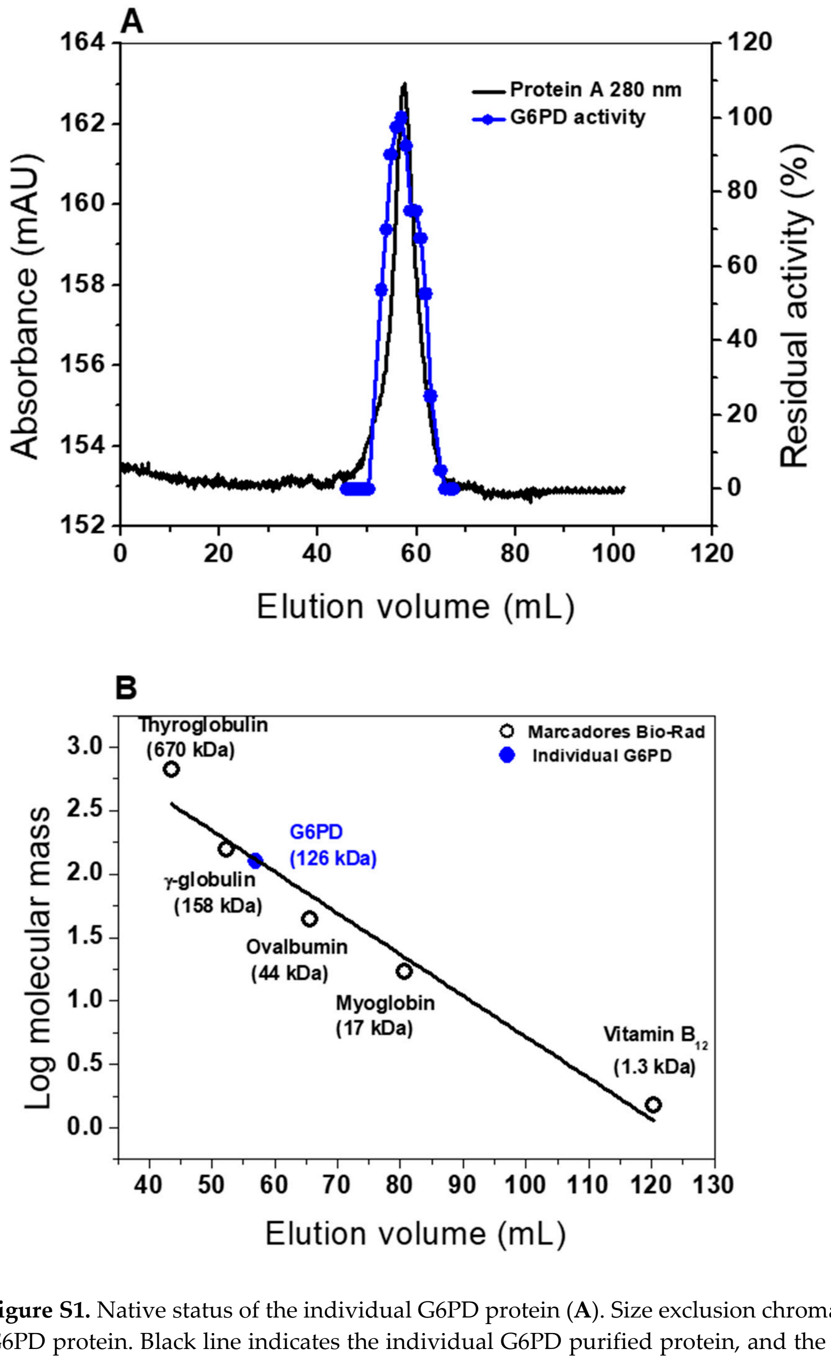

Figure S1. Native status of the individual G6PD protein (**A**). Size exclusion chromatography of individual G6PD protein. Black line indicates the individual G6PD purified protein, and the blue line represents the G6PD activity. (**B**) Calibration curve showing the elution volumes versus the log of the molecular weight (MW) of the Bio-Rad gel filtration standard (o). MW of the individual G6PD protein (●).
